# Influence of Preoperative Lumbar Deformity on Improvement in Low Back Pain Following Total Knee Arthroplasty

**DOI:** 10.7759/cureus.82544

**Published:** 2025-04-18

**Authors:** Takuma Maeda, Hiroshi Sasaki, Fumimasa Maruno, Ryosuke Kuroda, Tomoyuki Matsumoto

**Affiliations:** 1 Department of Orthopaedic Surgery, Kobe University Graduate School of Medicine, Kobe, JPN; 2 Department of Orthopedic Surgery, Kobe Rosai Hospital, Kobe, JPN

**Keywords:** knee-spine syndrome, oswestry disability index (odi), spinal sagittal alignment, total knee arthroplasty (tka), total knee replacement (tkr)

## Abstract

Purpose: Knee-spine syndrome emphasizes the interplay between knee and spinal alignments. Previous studies have reported that low back pain (LBP) is strongly associated with knee osteoarthritis (OA) pain. Therefore, we investigated how total knee arthroplasty (TKA) influences lumbar alignment and LBP, hypothesizing that LBP improves after TKA in patients without preoperative lumbar deformity.

Methods: We evaluated 87 patients undergoing unilateral primary TKA for knee OA. Lumbar sagittal alignment (C7 sagittal vertical axis (C7-SVA), pelvic tilt (PT), pelvic incidence (PI), lumbar lordosis (LL)), knee alignment (hip-knee-ankle angle), range of motion, and clinical outcomes (EQ-5D, Locomo, 2011 Knee Scoring System, Oswestry Disability Index (ODI)) were assessed preoperatively and at three and 12 months postoperatively. Patients were categorized into three clusters based on preoperative lumbar alignment (PI-LL) and changes in ODI.

Results: Radiographic changes included a significant increase in C7-SVA and LL, and a decrease in PT. Clinically, ODI and EQ-5D improved significantly at three months, with benefits persisting at one year. The greatest improvement in LBP was observed in a cluster with worse preoperative back pain but without severe lumbar deformity.

Conclusion: Patients without severe lumbar deformity but with LBP may benefit from TKA, as their LBP may be secondary to knee OA. In contrast, those with severe lumbar deformity may show limited LBP improvement postoperatively.

## Introduction

Knee-spine syndrome refers to the bidirectional relationship between knee osteoarthritis (OA) and spinal degeneration, wherein deterioration in one region can trigger compensatory changes and symptom exacerbation in the other [1-3]. Knee OA can negatively impact sagittal balance and lumbar spine loading, while spinal deformities may lead to abnormal gait patterns and increased stress on the knee joint [4]. Consequently, treatments addressing knee pathology, such as total knee arthroplasty (TKA), have the potential to affect not only knee function but also spinal alignment and related symptoms.

TKA significantly alters lower limb biomechanics, thereby influencing spinal sagittal alignment, potentially alleviating or exacerbating low back pain (LBP) [5,6]. However, the benefits of TKA on spinal alignment and symptoms largely depend on lumbar spine flexibility and preexisting spinal deformity severity [7]. Previous research indicates that knee pain severity is associated with LBP severity, suggesting potential concurrent benefits from knee-focused interventions [8-10]. However, severe lumbar deformities may limit or negate these benefits, whereas patients with relatively normal lumbar alignment could experience significant improvements in spine-related symptoms following TKA.

The relationship between spinal alignment and symptom improvement following TKA can be quantitatively assessed using sagittal parameters, such as pelvic incidence-lumbar lordosis (PI-LL) mismatch, pelvic tilt (PT), and the C7 sagittal vertical axis (C7-SVA), which reflect compensatory mechanisms in knee-spine syndrome [11-13]. Among these parameters, PI-LL mismatch is particularly significant, as it correlates strongly with the severity of spinal symptoms [13]. Despite existing research, the precise influence of preoperative lumbar alignment on postoperative spinal outcomes after TKA remains unclear.

This study aimed to evaluate how preoperative spinal alignment influences changes in pain, mobility, and sagittal alignment following TKA. We hypothesized that distinct subgroups exist based on lumbar deformity severity and corresponding LBP improvement after TKA, and identifying these subgroups could help clarify the spinal outcomes of knee-focused interventions.

## Materials and methods

Study design

**Figure 1:**
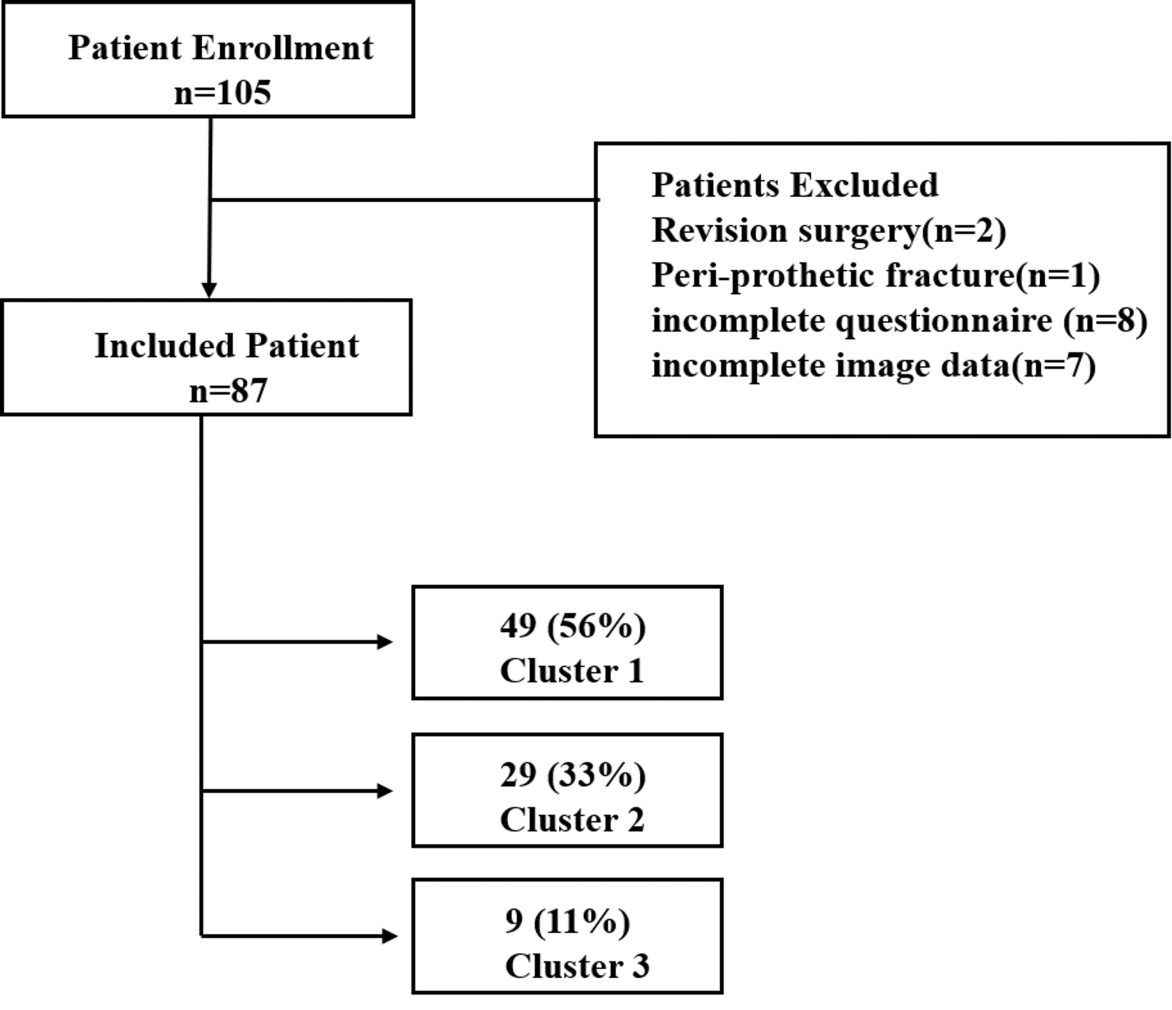
Patient enrollment and cluster classification Out of 105 consecutive cases, 87 patients meeting the inclusion criteria were analyzed. The patients were divided into three clusters based on the results of a cluster analysis. Figure Credits: T. Maeda (created using PowerPoint)

This retrospective study included patients who underwent unilateral primary TKA for knee OA between May 2019 and May 2021. Exclusion criteria were prior hip or spine surgery, postoperative lumbar or thoracic compression fractures during follow-up, and incomplete follow-up assessments. Ethical approval was obtained from the institutional review board, and informed consent was provided by all patients. Out of 105 consecutive cases initially identified, 87 met inclusion criteria (Figure 1).

Excluded cases consisted of two revision surgeries, one peri-prosthetic fracture, eight incomplete questionnaires, and seven incomplete imaging data. Patient demographics and preoperative characteristics are presented in Table 1.

**Table 1:** Patient characteristics and preoperative findings C7-SVA: C7-sagittal vertical alignment, HKA angle: hip-knee ankle angle, ROM: range of motion, SD: standard deviation

Characteristics n=87	Mean ± SD
Sex, N(％)	-
Male	18 (20.7)
Female	69 (79.3)
Age	76.7±7.2
Height (cm)	153.2±8.9
Weight (kg)	60.1±10.6
Body mass index (kg/m^2^)	25.5±3.3
follow-up time (months)	18.6±3.6
HKA angle (°)	10.4±7.3
C7-SVA (mm)	45.7±36.5
Pelvic tilt (°)	23.3±9.9
Pelvic incidence (°)	52.1±10.1
Lumbar lordosis (°)	45.5±12.1
ROM: extension (°)	-7.5±7.3
ROM: flexion (°)	120.9±11.9

Radiographic parameters

**Figure 2:**
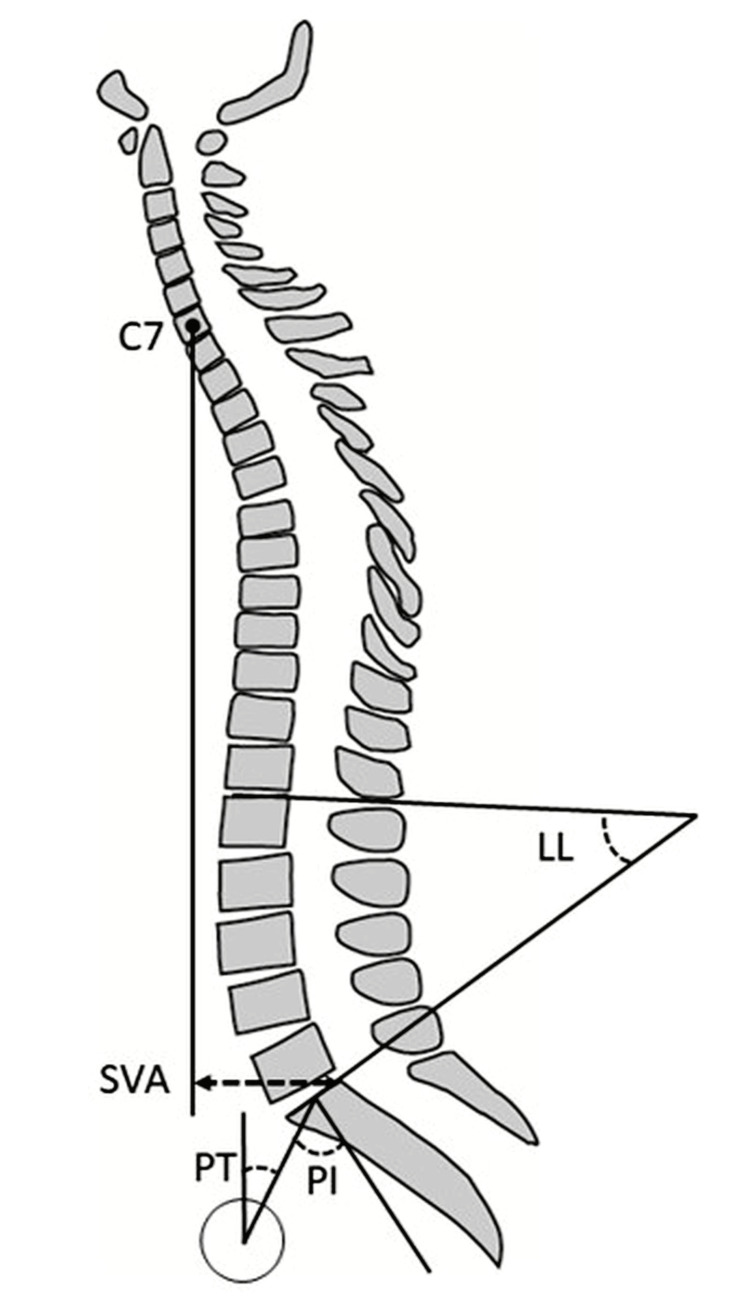
Parameters for evaluating spinal sagittal alignment C7-SVA: C7 sagittal vertical axis Positive values indicate anterior displacement, while negative values indicate posterior displacement, and it is measured in millimeters. PT: pelvic tilt, measured in degrees. PI: pelvic incidence, measured in degrees. LL: lumbar lordosis, measured in degrees. Figure Credits: H. Sasaki (created using Adobe Illustrator)

Spinal sagittal alignment parameters, including C7-SVA, PT, PI, and lumbar lordosis (LL), were measured from radiographs before surgery, three months postoperatively, and one year postoperatively [11,13] (Figure 2).

Knee alignment was evaluated using the hip-knee-ankle (HKA) angle, and knee range of motion (ROM), including flexion and extension, was recorded at the same time points.

Clinical outcomes

Clinical outcomes assessed preoperatively, at three months, and one year postoperatively included the Oswestry Disability Index (ODI) for evaluating LBP-related functional disability [14], EuroQol-5 Dimensions (EQ-5D) for quality of life [15], Locomo scale for locomotor function [16], and the 2011 Knee Society Score (KSS2011) for knee function, patient satisfaction, and expectations [17].

Cluster analysis

**Figure 3:**
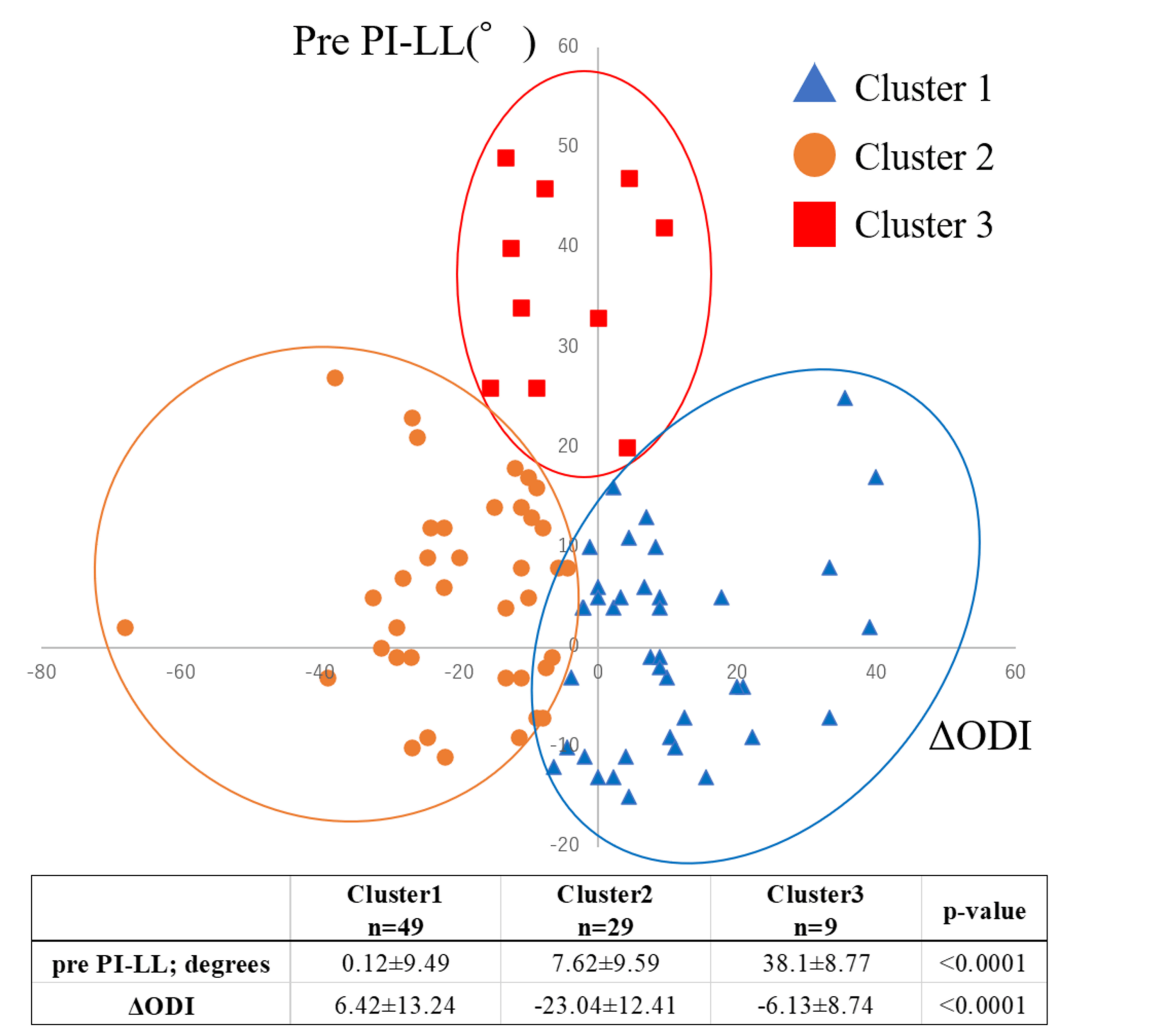
Preoperative pelvic incidence-lumbar lordosis mismatch and postoperative Oswestry Disability Index changes: cluster analysis of spinal alignment and functional outcomes Patients were divided into three clusters based on preoperative pelvic incidence-lumbar lordosis mismatch and difference in Oswestry disability index. Cluster 1 had normal lumbar alignment and no significant change in low back pain (LBP) after total knee arthroplasty. Cluster 2 also had normal lumbar alignment but experienced significant improvements in LBP postoperatively. Cluster 3, the smallest group, had severe lumbar deformity and showed no remarkable improvement in LBP. ODI: Oswestry Disability Index, PI-LL: pelvic incidence-lumbar lordosis mismatch

To identify distinct patient subgroups based on preoperative lumbar deformity and postoperative LBP outcomes, we conducted a cluster analysis using preoperative PI-LL mismatch, an established measure of lumbar deformity severity, and the change in ODI scores (ΔODI) as indicators of postoperative LBP improvement. PI-LL was selected as a widely validated sagittal modifier in adult spinal deformity classifications. ODI was chosen due to its reliability and extensive validation for assessing functional disability associated with LBP. Before clustering, both variables were standardized via z-score transformation to equalize weighting. We utilized the elbow method to determine the optimal cluster count, identifying three clusters. Patients were grouped using an unsupervised k-means clustering algorithm, categorizing individuals based on similarities in standardized PI-LL mismatch and ΔODI scores. Thus, clusters emerged naturally from the data structure rather than predefined thresholds. Patients were classified into three distinct clusters (Figure 3): Cluster 1 - Normal lumbar alignment without significant LBP improvement; Cluster 2 - Normal lumbar alignment with significant improvement; and Cluster 3 - Severe lumbar deformity with limited improvement.

Statistical analysis

Hierarchical cluster analysis was performed using Euclidean distance with EZR software to confirm patient groupings. Differences in clinical and radiographic outcomes within each patient group were assessed using repeated-measures ANOVA with Bonferroni correction. Inter-group differences among clusters were analyzed via one-way ANOVA, also employing Bonferroni correction. All statistical tests were two-sided, with a significance level set at p < 0.05. Interobserver and intraobserver interclass correlation coefficients (ICCs) were calculated, with ICC values for radiographic measurements exceeding 0.80.

## Results

Radiographic parameters and clinical outcomes

The C7-SVA increased significantly at three months postoperatively (mean: 8.5 mm, SD: 3.7, post-hoc p = 0.043) but slightly decreased at one year. The PT decreased significantly over time (mean: 1.3°, SD: 0.85, p < 0.001), indicating anterior tilting of the pelvis. Similarly, LL increased significantly (mean increase: 1.5°, SD: 0.6, p < 0.001). Knee ROM improved significantly in both flexion (mean: 3.9°, SD: 2.7, p = 0.012) and extension (mean: 6.3°, SD: 3.7, p < 0.001). Regarding clinical outcomes, the Oswestry Disability Index (ODI) improved significantly (p < 0.001), with a mean decrease of 5.9 points at three months (post-hoc p = 0.015). The EQ-5D and Locomo scores improved significantly over time (p < 0.001). The 2011 Knee Scoring System (KSS2011) score improved significantly over time (p < 0.001), except for the Expectation subscale, which decreased significantly (p < 0.001) (Table 2).

**Table 2:** Changes in parameters and clinical outcomes before and after total knee arthroplasty This table summarizes the changes in spinal alignment parameters (C7-SVA, PT, PI, and LL), range of motion (ROM: flexion and extension), and clinical outcomes (ODI, EQ-5D, Locomo, and KSS domains) at preoperative, three months postoperative, and one year postoperative time points. Positive values for C7-SVA indicate anterior displacement, and negative values indicate posterior displacement. p-values were calculated using repeated measures ANOVA to assess changes over time. Post-hoc comparisons were performed using Bonferroni correction for multiple testing. ODI: Oswestry Disability Index; EQ-5D: EuroQol-5 Dimensions; Locomo: Locomotive Syndrome Risk Test; KSS: 2011 Knee Scoring System

Parameters n=87	Preoperative	3-Month Postoperative	12-Month Postoperative	p-value (ANOVA)
C7-SVA (mm)	45.7 (36.5)	53.8 (37.2)	53.0 (40.2)	0.037
Pelvic tilt	23.3 (9.9)	22.9 (9.9)	22.1 (9.2)	<0.001
Pelvic incidence	52.1 (10.1)	52.1 (9.6)	52.5 (9.3)	0.841
Lumbar lordosis	45.5 (12.1)	46.0 (12.6)	47.3 (12.7)	<0.001
ROM: extension	-7.5 (7.3)	-3.8 (4.8)	-1.8 (3.5)	<0.001
ROM: flexion	120.9 (11.9)	121.3 (10.2)	124.6 (9.1)	0.012
HKA angle	10.4 (7.3)	0.9 (2.8)	0.7 (2.7)	<0.001
ODI	28.8 (16.8)	22.9 (16.2)	24.1 (16.7)	0.001
EQ5D	0.55 (0.21)	0.65 (0.25)	0.66 (0.22)	<0.001
Locomo	36.1 (18.0)	28.2 (16.9)	31.5 (21.2)	<0.001
KSS Satisfaction	15.5 (6.8)	21.4 (6.3)	23.4 (7.4)	<0.001
KSS Expectation	12.0 (2.8)	10.4 (3.5)	9.9 (3.2)	<0.001
KSS Function	46.8 (20.9)	60.5 (18.5)	57.5 (24.5)	<0.001
KSS Objective	45. 7 (19.5)	75.2 (26.1)	75.9 (25.3)	<0.001

The preoperative comparison across clusters is presented in Table 3.

**Table 3:** Comparison of characteristics, radiographic parameters, and clinical outcomes across clusters Cluster 1 (n=49) had normal lumbar alignment and no significant change in low back pain (LBP) after total knee arthroplasty. Cluster 2 (n=29) showed normal lumbar alignment with significant improvements in LBP postoperatively. Poorer preoperative clinical data and greater knee extension restriction were shown in Cluster 2. Cluster 3 (n=9) had severe lumbar deformity and showed no remarkable improvement in LBP. There were significant differences in preoperative lumbar alignment (C7-sagittal vertical axis, pelvic tilt, pelvic incidence, lumbar lordosis) as well as in knee extension angle. p-values were calculated using one-way ANOVA for continuous variables and chi-square test for categorical variables. Post-hoc comparisons were performed using Bonferroni correction for multiple testing. Of the variables compared among the three clusters, those with different superscript letters (a, b, or c) demonstrated statistically significant differences in the post hoc analysis. In contrast, variables without superscript letters did not reach statistical significance in the initial ANOVA or Kruskal–Wallis test. C7-SVA: C7-sagittal vertical axis, HKA angle: hip-knee ankle angle, KSS: 2011 Knee Scoring System, ODI: Oswestry Disability Index, ROM: range of motion

	Cluster 1 (n=49)	Cluster 2 (n=29)	Cluster 3 (n=9)	p-value
Sex, N(％)	-	-	-	-
Female	38 (77.6)	23 (79.3)	8 (88.9)	0.742
Male	11 (22.4)	6 (20.7)	1 (11.1)	-
Age	75.80 (7.39)	77.48 (6.71)	78.78 (7.22)	0.393
Height (cm)	154.35 (9.24)	151.64 (7.52)	151.49 (10.88)	0.363
Weight (kg)	60.90 (10.21)	58.76 (11.41)	59.07 (10.66)	0.67
BMI (kg/m^2^)	25.50 (3.09)	25.43 (3.73)	25.68 (3.26)	0.98
C7-SVA (mm)	35.86 (27.25)^a^	43.22 (27.57)^a^	106.78 (49.01)^b^	<0.001
Pelvic tilt (°)	19.80 (8.08)^a^	23.55 (7.20)^a^	41.22 (6.69)^b^	<0.001
Pelvic Incidence (°)	48.61 (8.80)^a^	54.69 (9.90)^b^	62.33 (8.00)^c^	<0.001
Lumbar Lordosis (°)	48.49 (9.12)^a^	47.07 (10.59)^a^	24.22 (10.47)^b^	<0.001
ROM: extension (°)	-5.61 (5.74)^a^	-8.28 (7.47)^a^	-15.56 (8.82)^b^	<0.001
ROM: flexion (°)	121.29 (11.39)	121.38 (12.31)	117.22 (13.72)	0.623
HKA angle (°)	10.27 (7.31)	10.08 (8.02)	11.00 (7.32)	0.562
ODI	21.20 (13.91)^a^	39.15 (14.90)^b^	37.02 (16.66)^b^	<0.001
EQ5D	0.61 (0.18)^a^	0.48 (0.24)^b^	0.48 (0.21)^b^	0.021
Locomo	32.23 (16.27)	40.96 (18.32)	41.15 (22.58)	0.077
KSS Satisfaction	15.84 (6.10)	14.89 (6.96)	15.83 (9.73)	0.828
KSS Expectation	12.01 (3.29)	11.95 (1.96)	12.31 (2.43)	0.946
KSS Function	49.39 (19.46)	43.57 (21.40)	42.97 (26.61)	0.421
KSS Objective	49.76 (18.79)	41.54 (19.72)	36.56 (20.23)	0.068

There were no significant differences in background data across clusters. However, all sagittal spinal alignment parameters (C7-SVA, PT, LL) showed significant differences across clusters (p < 0.001), with Cluster 3 exhibiting worse alignment compared to Clusters 1 and 2. While the HKA angle and ROM flexion showed no significant differences across clusters, ROM extension was significantly worse in Cluster 3 compared to Clusters 1 and 2 (post-hoc p < 0.001). Additionally, ODI and EQ-5D scores were significantly worse in Clusters 2 and 3 compared to Cluster 1 (post-hoc p < 0.001 for both). The preoperative KSS objective score tended to be worse in Cluster 3.

Changes between preoperative and one-year postoperative across clusters are presented in Table 4. Changes in sagittal spinal alignment were similar across clusters. While Cluster 3 showed the greatest improvement in ROM extension compared to Cluster 1 (post-hoc p = 0.002) and Cluster 2 (post-hoc p = 0.086), Cluster 2 showed greater improvements in most clinical scores, such as ODI, EQ-5D, and Locomo, demonstrating significantly better outcomes than Cluster 1 (post-hoc p < 0.001) and favorable, though not statistically significant, differences compared to Cluster 3.

**Table 4:** Comparison of changes in radiographic parameters and clinical outcomes between preoperative and one-year postoperative across clusters The knee extension angle showed the most significant improvement in Cluster 3. Meanwhile, Cluster 2, which experienced the greatest improvement in ODI, demonstrated the most significant improvements in EQ5D and Locomo. p-values were calculated using one-way ANOVA for continuous variables. Post-hoc comparisons were performed using Bonferroni correction for multiple testing. Of the variables compared among the three clusters, those with different superscript letters (a, b, or c) demonstrated statistically significant differences in the post-hoc analysis. In contrast, variables without superscript letters did not reach statistical significance in the initial ANOVA or Kruskal–Wallis test. *Δ represents the difference in parameters between preoperative and 1-year postoperative values.

	Cluster 1 (n=49)	Cluster 2 (n=29)	Cluster 3 (n=9)	p-value
*ΔC7-SVA (mm)					3.48 (28.14)	12.01 (27.46)	13.18 (64.70)	0.473
ΔPelvic tilt (°)	-0.87 (3.33)	-1.44 (4.65)	-2.11 (6.90)	0.671
ΔLumbar lordosis (°)	1.21 (8.54)	1.78 (5.20)	5.22 (7.29)	0.338
ΔROM: extension (°)	3.90 (5.61)^a^	6.78 (7.00)^a^	12.22 (8.33)^b^	0.002
ΔROM: flexion (°)	2.90 (11.20)	3.74 (9.21)	7.78 (10.64)	0.445
ΔHKA angle (°)	-9.60 (4.72)	-9.73 (4.90)	-10.11 (5.41)	0.297
ΔODI	6.42 (13.24)^a^	-23.04 (12.41)^c^	-6.13 (8.74)^b^	<0.001
ΔEQ5D	0.03 (0.26)^a^	0.22 (0.23)^b^	0.16 (0.09)^a^	0.004
ΔLocomo	-0.83 (15.48)^a^	-10.95 (14.06)^b^	-3.87 (15.08)^a^	0.019
ΔKSS Satisfaction	6.89 (9.39)	9.53 (7.55)	7.87 (7.03)	0.427
ΔKSS Expectation	-2.54 (4.02)	-1.37 (3.19)	-2.20 (4.20)	0.422
ΔKSS Function	8.10 (25.46)	16.47 (19.84)	6.02 (17.59)	0.251
ΔKSS Objective	24.72 (28.97)	36.59 (25.07)	39.44 (31.50)	0.119

## Discussion

The initial findings of our study illustrate that TKA is associated with significant changes in spinal sagittal alignment and impacts LBP. These changes include decreased PT, increased LL, and an anterior shift in the center of gravity, as indicated by an increase in the C7-SVA, a marker of spinal sagittal alignment. These findings suggest that improvements in knee flexion contractures allow for greater knee extension, resulting in anterior pelvic tilt and increased SVA. Consequently, LL increases compensations, consistent with previous literature [11,18,19]. However, the degree of improvement in LBP after TKA observed in our results is relatively modest, falling short of the minimum clinically important difference of 12.8 points [20], suggesting that outcomes may vary depending on individual cases.

The concept of knee-spine syndrome highlights the interdependence between knee OA and spinal alignment or symptoms, where dysfunction in one area can adversely affect the other. Previous studies have shown that knee OA can contribute to LBP through altered gait and posture [21], such as reduced knee extension during the standing position [5], increased anterior pelvic tilt [22], and compensatory trunk lean to avoid knee pain [23]. These biomechanical adaptations may increase spinal loading and accelerate lumbar degeneration or muscular fatigue, ultimately leading to LBP.

Previous studies have shown that LBP is strongly associated with knee pain due to knee OA [8], and patients with severe LBP report significantly worse WOMAC pain scores than those with mild symptoms [10]. A recent systematic review by Amarasinghe et al. further emphasized the high co-occurrence of LBP and knee OA, with greater pain intensity, disability, and fall risk [24]. However, it remains unclear whether LBP or knee pain arises first. Based on these findings, we hypothesized that LBP originating from spinal degeneration differs from that caused by knee OA. To explore this distinction, we conducted a cluster analysis using PI-LL and ODI. While previous studies have independently linked PI-LL mismatch to worse ODI scores [13,25], we combined these parameters to identify clinically meaningful subgroups. Our initial analysis using PI-LL and absolute ODI failed to reveal clear patterns, likely due to variability in baseline ODI scores. Replacing ODI with ΔODI (change from pre- to postoperative) allowed better differentiation of patient subgroups and clarified the clinical implications of our results.

The key takeaway is that patients in Cluster 3, who exhibited severe lumbar deformity with no remarkable improvement in LBP, are likely experiencing LBP due to spine-originated issues. Although this group had the greatest improvement in knee extension angle, their severe LBP did not improve. Furthermore, this group showed significantly worse preoperative sagittal spinal alignment, a characteristic often observed in patients with adult spinal deformity, suggesting a breakdown of the spinal compensatory mechanisms [13]. This may indicate that LBP arising from lumbar degenerative diseases does not improve after TKA.

In contrast, patients in Cluster 2, who had normal lumbar alignment with significant postoperative improvements in LBP, likely experienced LBP originating from knee OA. Iijima et al. reported that the varus thrust, which frequently occurs in knee OA, is a factor associated with more severe knee OA-originated LBP [23]. These authors suggested that proximal gait adaptations, such as lateral trunk leaning to avoid knee pain, may result in muscle soreness and fatigue, leading to myofascial LBP. Furthermore, Ramsook et al. noted that myofascial LBP arises from muscular dysfunction, differing from LBP caused by spinal degeneration [26]. Correcting the knee pathology in these patients through TKA may greatly improve LBP.

This study provides valuable insights into the relationship between lumbar alignment, TKA, and LBP, but there are several limitations to consider. Firstly, the small sample sizes, especially in Cluster 3, reduce statistical power and may obscure subtle outcomes. Secondly, this study included only patients who underwent unilateral TKA. The presence of flexion contracture or other deformities in the contralateral knee, which were not addressed in this study, may have influenced sagittal alignment and LBP improvement. Additionally, other potential spinal pathologies, such as scoliosis, muscle imbalance, or early disc degeneration, were not systematically assessed and could have influenced postoperative outcomes. While validated tools such as the ODI and EQ-5D were used to evaluate LBP and quality of life, their applicability to this specific patient population may be limited and should be interpreted with caution. Moreover, as this was a single-center study, future multicenter studies with larger cohorts are warranted to validate the generalizability of our findings. Finally, the short follow-up period may not fully capture the long-term effects on LBP and functional outcomes.

## Conclusions

Our findings suggest that patients with no significant lumbar spine deformity but who suffer from severe LBP, such as those in Cluster 2, may have experienced myofascial or other forms of secondary LBP related to knee OA and may therefore benefit substantially from TKA. In contrast, patients with severe lumbar deformity, like those in Cluster 3, experience minimal improvement in LBP post-surgery due to lumbar-originated issues. These results, based on the novel perspective of distinguishing LBP into spine- and knee-originated causes, highlight the originality of this study and suggest substantial treatment benefits through tailored approaches to individual patient conditions. These findings highlight the importance of assessing PI-LL mismatch prior to TKA, as it may help guide surgical sequencing, optimize candidate selection, and refine expectations for LBP improvement. However, further follow-up and larger-scale studies are needed to validate these findings and fully understand their clinical implications.
